# Maternal–Fetal Outcomes in Women with Endometriosis and Shared Pathogenic Mechanisms

**DOI:** 10.3390/medicina57111258

**Published:** 2021-11-17

**Authors:** Francesca Frincu, Andreea Carp-Veliscu, Aida Petca, Dumitru-Cristinel Badiu, Elvira Bratila, Monica Cirstoiu, Claudia Mehedintu

**Affiliations:** 1 Department of Obstetrics and Gynecology, “Carol Davila” University of Medicine and Pharmacy, 020021 Bucharest, Romania; frincu.francesca@gmail.com (F.F.); aidapetca@gmail.com (A.P.); elvirabarbulea@gmail.com (E.B.); dr_cirstoiumonica@yahoo.com (M.C.); claudiamehedintu@yahoo.com (C.M.); 2 Department of General Surgery, “Carol Davila” University of Medicine and Pharmacy, 020021 Bucharest, Romania; doctorcristianbadiu@yahoo.com

**Keywords:** endometriosis, obstetric, complications, preeclampsia, hemorrhages, miscarriage, stillbirth

## Abstract

The connection between endometriosis and pregnancy outcomes is trending among the research topics. Until recently, endometriosis and its painful symptomatology were considered to be alleviated by pregnancy. However, these beliefs have shifted, as emerging literature has demonstrated the role of this condition in affecting pregnancy evolution. The underlying pathogenesis of endometriosis is still poorly understood, all the more when pregnancy complications are involved. Debatable opinions on endometriosis associated with obstetric complications exist because of the potential bias resulting from the heterogeneity of preceding evidence. This review aims to evaluate the connection between endometriosis and adverse pregnancy outcomes and their shared pathogenic mechanisms. We searched PubMed and EMBASE and focused on the studies that include placenta praevia, premature rupture of membranes, spontaneous preterm birth, gestational hypertension, preeclampsia, obstetric hemorrhages (ante- and postpartum bleeding, abruptio placentae), miscarriage, stillbirth, neonatal death, gestational diabetes mellitus, gestational cholestasis, small for gestational age, and their association with endometriosis. Not only the risks of emergence were highlighted, but also the pathogenic connections. Epigenetic alterations of some genes were found to be mirrored both in endometriosis and obstetric complications. This review issues a warning for providing increased attention to pregnant women with endometriosis and newborns as higher risks of preeclampsia, placental issues, and preterm deliveries are associated.

## 1. Introduction

Endometriosis is a debilitating disease that occurs in 10 to 15% of the general population and is present in 30–50% of women with infertility [1,2]. It is considered a condition of premenopausal women. The peak of prevalence shows coincidence with the female reproductive period [3]. The main complaints are dysmenorrhea, dyspareunia, and pelvic pain. Symptoms from the gastro-intestinal area and infertility are also common and interfere with social, mental, and physical well-being [4,5]. It is estimated that the cost of the disease, in terms of loss of work productivity and illness, is over $9911 per patient per year, causing a substantial economic burden [6].

The disease is defined by the presence of endometrial-like tissue (stroma and glands) outside the uterine cavity, distorting the pelvic anatomy through inflammatory reaction, adhesions, and scar tissue [7,8]. The still debatable pathogenesis of endometriosis raises a challenge for doctors in predicting the natural evolution, possible complications, and correct management. Endometriosis displays three phenotypes, superficial peritoneal lesions (SUP), ovarian endometrioma (OMA), and deep endometriosis (DE). DE represents the most severe phenotype, defined as lesions that penetrate under the peritoneal surface deeper than 5 mm such as the uterosacral ligaments, the muscularis propria of the bladder, the ureter with or without ureterohydronephrosis, and intestine with or without occlusion [9]. Extra-pelvic endometriosis is reported on the pleural, umbilical, neural, and diaphragmatic levels [10]. Some indicators for the disease extension may help doctors acquire the ‘big picture’: DE is considered the ‘abdominal-pelvic multifocal disease’ rather than a unique organ pathology. The DE nodules would rather present multifocal distribution than isolated [9]; OMA expresses more severe DE [11,12]. The endometrial tissue infiltration in the myometrium characterizes diffuse and focal adenomyosis [13,14,15]. Adenomyosis is regularly associated with endometriosis but contributes independently to infertility [16,17], bleeding (menorrhagia and metrorrhagia) [18,19], and pain [20].

The most important theories about endometriosis pathogenicity date back to 1927 and 1942. Peritoneal seeding through retrograde menstruation was first described by Sampson [21]. Gruenwald later proposed the metaplasia of the mesothelial cell [22,23]. More factors are thought to contribute to the pathogenicity such as impaired steroid biosynthesis (aromatase overexpression, hyperestrogenism, progesterone resistance) [24,25], dysregulated immunity [26,27,28], inflammatory [29], environmental, genetic, and epigenetic factors [30,31], all acting in unison to determine endometriosis. Apart from the well-investigated complications, the literature also reports multiple comorbidities that may arise during the natural course of this illness. Chronic diseases such as pelvic inflammatory diseases, irritable bowel syndrome, migraines, chronic liver disease, chronic renal disease, diabetes mellitus, autoimmune diseases, obesity, hypertension, cardiovascular diseases, and hyperlipidemia have been the most highlighted in studies [32,33]. Malignant conditions such as gynecological cancers (endometrial, ovarian, breast), colorectal, and even thyroid [34,35,36] have also been linked to endometriosis.

Recently, researchers have focused on the increased risk of pregnancy complications. Due to endometriosis-associated infertility, many women resort to assisted reproductive technology (ART) to conceive, increasing the risk of adverse pregnancy outcomes [28]. The underlying pathogenesis that links endometriosis with pregnancy complications is still poorly known. The purpose of this review is to evaluate the connection between endometriosis and adverse pregnancy outcomes and their shared pathogenic mechanisms.

Controversial opinions on endometriosis involvement in obstetric complications exist [37,38] because of potential bias resulting from the heterogeneity of previous studies.

## 2. Materials and Methods

PubMed and EMBASE were searched from their inception until 10 October 2021 for all studies on endometriosis and adverse pregnancy outcomes. We focused our search on pathogenic mechanisms, placenta praevia, premature rupture of membranes, spontaneous preterm birth, gestational hypertension, preeclampsia, obstetric hemorrhages (ante- and postpartum bleeding, abruptio placentae), miscarriage, stillbirth, neonatal death, gestational diabetes mellitus, gestational cholestasis, and small for gestational age. The reference lists of the included studies were also screened for additional literature.

We used “MeSH” (PubMed) and “Emtree” (EMBASE) terms, but also free text words. We included cohort studies, case-control studies, systematic reviews, and meta-analyses. Case reports, case series, letters, conference abstracts, editorials and commentaries were excluded. Studies in another language than English were excluded.

## 3. Pathogenic Mechanisms Involved in Adverse Pregnancy Outcomes in Women with Endometriosis

Endometriosis induces local and systemic cytokine expression changes that disrupt the primary endometrial function [39]. Women’s endometrium presents an induction of p450 aromatase expression, changing the dynamic of progesterone to estrogen activity and promoting growth and endometriosis development [40]. Moreover, inflammation is known to influence steroid receptor and aromatase expression [41]. The enhanced local production of estrogen was shown to inhibit ανβ3 integrin, which plays a key role in the attachment of the embryos with consequent lower IVF (in vitro fertilization) outcomes [42,43]. Leukemia inhibitory factor (LIF), a pro-implantation cytokine, also has a reduced expression in the affected endometrium [44]. The L-selectin ligand contributes to embryo attachment and is decreased in unexplained infertility and endometriosis [45]. Maternal systemic inflammation with consequent environmental insult has been proposed to cause preeclampsia and preterm birth [46]. The peritoneal fluid in women with endometriosis has a whole change of cytokine milieu. It is abundant in interleukin-17 (IL-17), stimulating cyclooxygenase-2 (Cox-2) activity and IL6, IL-8, and aromatase expression [47]. Progesterone receptors are decreased in the endometrium. There is a downstream effect of progesterone with a more proliferative endometrium [48,49]. Estrogen receptors 1 (ER1) are usually downregulated in the secretory phase at the moment of implantation. Still, in endometriosis, there is a deficit in this phenomenon, and implantation failure can be easily predicted [50]. The shift to estrogen dominance generates factors that promote immunosuppression, angiogenesis, inflammation, and cell proliferation. Typically, progesterone triggers the endometrium to a response that induces and prolongs embryo implantation, called decidualization. The decidua is of utmost importance for the maternal/embryo interface. It protects the embryo from immune rejection and stress pathways, providing nutrients and modulating trophoblast invasion. Progesterone resistance in endometriosis is one of the essential elements that impair embryo implantation, mainly through a pro-inflammatory condition. Normally, it decreases inflammation in the endometrium and reversely inhibits uterine muscle contractility [24,51].

Emerging evidence shows that epigenetics and genetics play a critical role in the aberrant modification in women with endometriosis [24,51,52]. Abnormal DNA methylation and histone modification have been linked to an increased risk of endometriosis and reproductive disorders such as excessive gestational weight gain and maternal obesity [53]. The initial stage of development is a multistep process resulting from a sequential accumulation of epigenetic and genetic abnormalities, and the following is progression [54]. Epigenetic factors via DNA-methylation also contribute to progesterone resistance, as shown by Li et al. [55]. In the first stage, the Homebox A gene cluster (HOXA), an endometrium-related disease-specific methylation pattern, is responsible for initiation in the development of endometriosis lesions [56]. Some decidualization-related genes are downregulated in both the endometriotic lesions and endometrium of women with endometriosis—HOXA10, forkhead box O1 (FOXO1), Indian hedgehog signaling molecule (IHH), and CCAAT/enhancer-binding protein-beta (C/EBPbeta) [57,58]. Kobayashi et al. indicated that there are some shared genes, both paternally (DIRAS family GTPase 3—DIRAS3, cytochrome P450 family 1 subfamily B member 1—CYP1B1, Insulin-like growth factor 2—IGF2, bone morphogenetic protein 8b—BMP8B, zinc finger and AT-hook domain containing—ZFAT) and maternally (fibroblast growth factor receptor-like—FGFRL1, disheveled segment polarity protein 1—DVL1, H19, cyclin-dependent kinase inhibitor 1C—CDKN1C) that are common for endometriosis and obstetric complications such as preeclampsia, failure in decidualization process, pregnancy loss, small for gestational age (SGA), and preterm loss [59]. The progression comprises all changes induced by the oxidative stress and methylation errors that are generated in the affected cells. Autooxidation and Fenton’s reaction result in the formation of superoxide radicals and other reactive oxygen species (ROS) due to retrograde menstruation with consequent heme, hemoglobin, and iron [60,61]. ROS exposure determines and increases DNA methyltransferases (DNMTs) with consequent downregulation of the decidualization genes [61]. Researchers showed that distal-less homeobox 5—DLX5 (role in tissue damage repair) and GATA binding protein 3 (GATA3) (associated with cell biology), both paternally imprinted genes, were dysregulated in placenta tissues from women with preeclampsia and endometriosis [62,63,64]. Cyclin-dependent kinase inhibitor 1C (CDKN1C) (regulator of cell proliferation and cell cycle regulation), a maternal growth inhibitor gene, was dysregulated in preeclampsia and upregulated in the SGA placenta [62,65]. Hypomethylation of prostaglandin E receptor 2 (PTGER2), a crucial regulator of early pregnancy, was attributed to preterm birth [66].

**Figure 1:**
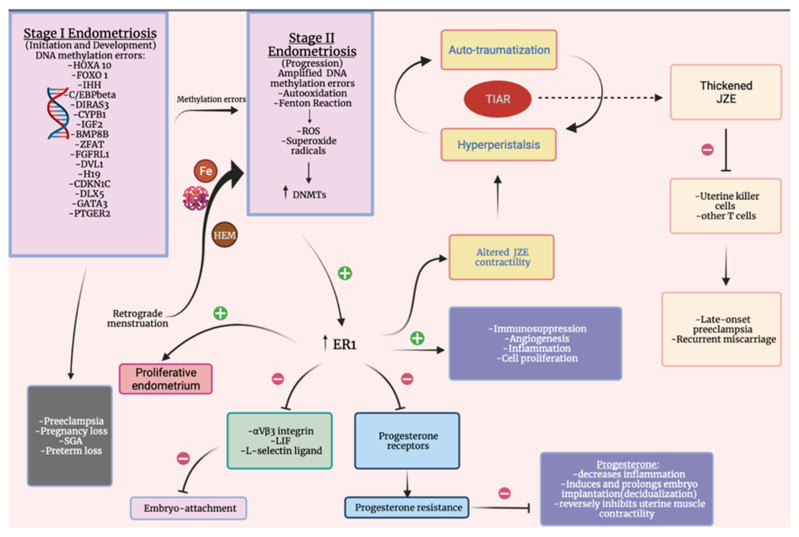
Representation of pathogenic mechanisms; HOXA—Homebox A gene cluster, FOXO1—forkhead box O1, IHH—Indian hedgehog signaling molecule, C/EBPbeta—CCAAT/enhancer-binding protein-beta, DIRAS3—DIRAS family GTPase 3, CYP1B1—cytochrome P450 family 1 subfamily B member 1, IGF2—Insulin-like growth factor 2, BMP8B—bone morphogenetic protein 8b, ZFAT—zinc finger and AT-hook domain containing, FGFRL1—fibroblast growth factor receptor-like, DVL1—disheveled segment polarity protein 1, H19, CDKN1C—cyclin-dependent kinase inhibitor 1C, DLX5—distal-less homeobox 5, GATA3—GATA binding protein 3, SGA—small for gestational age, DNMTs—DNA methyltransferases, ROS—reactive oxygen species, LIF—Leukemia inhibitory factor, PTGER2—prostaglandin E receptor 2, TIAR—tissue injury and repair, JZE—junctional zone endometrium.

The junctional zone endometrium (JZE) appears extended, thickened, disrupted, and has altered peristalsis in severe endometriosis and adenomyosis [67]. Adenomyosis has been associated with an increased risk for preterm premature membrane rupture (PPROM) and spontaneous preterm delivery [68]. Incomplete or absent remodeling of spiral arteries during pregnancy within the JZE can cause obstetric complications such as placental abruption, preterm birth, second-trimester miscarriage, preeclampsia, and fetal growth restriction (FGR) due to deep placentation [69]. The altered JZE contractility may reside from hyperestrogenism, causing a vicious cycle of hyperperistalsis and auto-traumatization, explaining the tissue injury and repair (TIAR) pathological theory [70]. Late-onset preeclampsia associates high thickness of JZEmax (9 mm versus 13 mm; *p* < 0.005). Additionally, recurrent miscarriages were linked to increased thickness JZEmax (5 mm; *p* < 0.01) [71]. Uterine killer cells and other T cells play an important role in normal decidualization including remodeling myometrial spiral arteries from the JZE. Endovascular trophoblast invasion is impeded by the lack of natural killer cells inside the thickened JZE [72]. In the non-pregnant state, in the early follicular phase right after menstruation ‘endometrial waves’, the contractions involving the sub-endometrial layer in the myometrium last 10–15’ and occur once or again twice in a minute. Implantation occurs due to a decrease in amplitude and frequency of the contractions during the luteal phase and then dramatically increase to labor-like contractions during menstruation [73]. Studies show that uterine contractions in women with endometriosis have higher basal pressure tone, frequency, and amplitude [74]. The common pathogenic mechanisms in endometriosis and obstetric complications are represented in Figure 1.

## 4. Placenta Praevia

Placenta praevia (PP) was demonstrated to be associated with endometriosis and adenomyosis due to impaired peristalsis that harms the potential location of blastocyst implantation. Vercellini et al. showed that the incidence of PP in 150 women with rectovaginal endometriotic lesions was 7.6%, compared to 2.1% in 69 women with ovarian and peritoneal implants group and 2.4% in 100 women with only peritoneal implants. The risk for placental anomalies was six times higher for women with rectovaginal lesions [75]. Unfortunately, few studies firmly attest to the connection between PP and endometriosis. It is for sure that ART procedures do increase the risk for PP, and endometriosis patients have higher achievements of pregnancy through ART. Whether endometriosis itself or ART predisposes to adverse pregnancy outcome including PP remains a debatable subject. A meta-analysis conducted by Gasparri et al. shed light on this matter and revealed that endometriosis is a potentially independent and additional risk factor of PP. The outcomes following ART were different when comparing the endometriosis patients, with a threefold higher risk for PP (OR 2.96, 95% CI, *p* = 0.01), than women without endometriosis [76]. Similarly, Tatsuya et al. showed that PP was more frequent in women laparoscopically diagnosed with endometriosis (OR 12.1; 95% CI, 3.6–41.1) [77]. Lin et al. conducted a study on a cohort of 249 women with endometriosis and also found an elevated risk of PP in this group (adjusted OR 4.51; 95% CI, 1.23–16.50, *p* = 0.023) [78]. Kmietowicz Zosia used data from all NHS hospitals in Scotland and evaluated the pregnancy outcomes from 5375 women with endometriosis. He concluded that PP had a twofold higher risk of appearing in women with this condition [79]. Lalani et al. concluded in a meta-analysis that for the subgroup of women with endometriosis who conceived spontaneously, the OR (95% CI) for PP was 6.83, for those who conceived through ART—3.33, and those with combined spontaneous and ART pregnancy—3.31 [80]. Finally, Takemura et al. analyzed 318 ART pregnancies and found an OR of 15.1 in women with endometriosis [81].

## 5. Premature Rupture of Membranes, Spontaneous Preterm Birth

Premature rupture of membranes (pPROM) is the rupture of membranes before 37 weeks of gestation. Spontaneous preterm birth (PTB) is defined as spontaneous labor before 37 completed weeks of gestation followed by live birth. Metalloproteinases (MMP-9) and collagenases are implicated in collagen degradation within fetal membranes due to physical/microbial inflammatory circumstances that are highly observed in women with endometriosis at first pregnancy. The high levels of PGs and inflammatory cytokines that exist in endometriosis locally activate MMP-9 and determine the lesions’ invasiveness [82,83]. Conti et al. found that at primiparas with endometriosis, the OR for pPROM and PTB was 2.93 (*p* = 0.001) and 2.24 (*p* = 0.0005), respectively [84]. On a large cohort of 11,739 Danish women with endometriosis, Berlac et al. observed that OR for pPROM was 1.3 (95% CI, 1.1–1.5), for birth before 28 weeks of gestation 3.1 (95% CI, 2.7–3.6) and birth before 34 weeks 2.7 (95% CI, 2.5–2.9) [85]. Similarly, Lalani et al. reported in an extensive meta-analysis that the neonates and fetuses of women with endometriosis were more likely to have pPROM (OR = 2.33 [1.39–3.90]) [80]. Stephansson et al. studied a cohort of 8922 women diagnosed with endometriosis before delivery and concluded that preterm birth was higher in women with this condition compared to those without endometriosis (OR 1.33; 95% CI, 1.23–1.44). Moreover, women with endometriosis who conceived with ART had a risk for preterm birth of 1.24 (95% CI, 0.99–1.54) compared to those who conceived spontaneously, with a risk of 1.37 (1.25–1.50) [86].

## 6. Gestational Hypertension, Preeclampsia

Pregnancy-induced hypertension (PIH) appears in 6–10% of pregnancies. It is defined as systolic blood pressure (SBP) above 140 mmHg and diastolic blood pressure above (DBP) above 90 mmHg. PIH can refer to four distinct conditions, but we will only discuss gestational hypertension and preeclampsia in this review. PIH is considered an important cause of fetal, newborn, and maternal morbidity and mortality. Mothers with PIH present a greater risk for cerebrovascular events, disseminated intravascular coagulation, abruptio placentae, and organ failure. These women’s fetuses are at greater risk for prematurity, intrauterine growth retardation, and intrauterine death [87]. The first to propose a potential connection between endometriosis and preeclampsia were Kortelahti et al. in 2003, with no significant correlation [88]. A large Swedish study conducted by Stephansson et al. in 2009 observed an increased risk for preeclampsia in women with endometriosis (adjusted OR = 1.13, 95% CI 1.02–1.26; *p* = 0.05) [86]. Lalani et al. also evoked in a meta-analysis that endometriosis associates with higher risks for preeclampsia (13 studies; OR = 1.18 [1.01–1.39]) and gestational hypertension and/or pre-eclampsia (24 studies; OR = 1.21 [1.05, 1.39]) [80]. In a 2021 meta-analysis, Breintoft et al. identified 14 cohort studies analyzing endometriosis and gestational hypertension and 21 cohort studies regarding endometriosis and preeclampsia [37]. The results concluded that women with endometriosis were at greater risk for gestational hypertension (OR: 1.14 (95% CI: 1.00–1.31) and preeclampsia (OR: 1.19, 95% CI: 1.08–1.31) [37]. Berlac et al. also found that the OR for preeclampsia was 1.5 (95% CI, 1.3–1.5) and for severe preeclampsia, eclampsia, and HELPP 1.7 (95% CI, 1.5–2.0) [85]. Subsequent studies did not find an association between endometriosis and preeclampsia [75,78,84,89].

## 7. Obstetric Hemorrhages (Ante- and Postpartum Bleeding, Abruptio Placentae)

Placental abruption (PA), also called abruptio placentae, occurs due to compromise of the vascular structures that support the placenta and represents the early separation of the placenta from the lining of the uterus before completion of the second stage of labor. The vascular network is disrupted when the vascular structure is compromised because of substance use, hypertension, or conditions that cause the stretch of the uterus such as trauma [90]. Antepartum hemorrhage (APH) is defined as bleeding from the canal birth of 15 mL or more, after 20 weeks of gestation, before the baby’s birth. Postpartum hemorrhage (PPH) is defined as bleeding more than 500 mL within 24 h of vaginal delivery and greater than 750 mL after Caesarean section [91]. Horton et al. conducted an extensive meta-analysis showing that women with endometriosis that conceived either spontaneously or with ART have an 87% risk for PA (OR 1.87, CI 1.65–2.13, *p* < 0.001; *n* = 8) [92]. Similarly, Berlac et al. identified an increased risk for PA with an OR of 2.0 (95% CI, 1.7–2.3) and for antepartum hemorrhage after 22 weeks of gestation with an OR of 2.3 (95% CI, 2.0–2.5), but the risk for postpartum hemorrhage was slightly decreased compared to women without endometriosis [85]. Stephansson et al. found that the risk for antepartum bleeding including placental disorders was almost 80% (OR 1.76; 95% CI, 1.56–1.99) [86]. Healy et al. conducted a study on over 6730 births to determine the prevalence and risk factors for obstetric hemorrhage. They stated that endometriosis raises the risk not only for APH (OR 1.2; 95% CI, 0.95–1.53), but also for PPH (OR 1.28; 95% CI, 1.06–1.56) [91]. Breintoft et al. found an increased risk for PA with a pooled OR of 1.40 (95% CI: 1.12–1.76) and PPH of 1.05 (95% CI: 0.93–1.19) [37].

## 8. Miscarriage, Stillbirth, and Neonatal Death

In recent years, increased concern has been focused on miscarriages caused by endometriosis and adenomyosis. Results from studies remain quite controversial since some studies have presented negative results and others have reported positive results. In 2020, Huang et al. published a meta-analysis that investigated the miscarriage risk according to the mode of conceiving (spontaneous vs. ART), the endometriosis stage (according to rASRM: I/II vs. III/IV), and endometriosis type (SUP/OMA/DE). Miscarriage was defined as spontaneous abortion before 28 weeks of gestation. The risk of miscarriage for women with endometriosis was increased for those who conceived spontaneously (OR: 1.81, 95% CI: 1.44–2.28, *I*^2^ = 96%), and for those who were previously diagnosed with adenomyosis and conceived with ART, the risk was much higher (OR: 2.81, 95% CI: 1.44–5.47, *I*^2^ = 64%) [93]. Moreover, compared with women without endometriosis, women with SUP (OR: 2.01, 95% CI: 1.22–3.31, *I*^2^ = 75%) and women with DE (OR: 1.55, 95% CI: 1.20–2.02, *I*^2^ = 0%) had higher miscarriage risk, while unresected OMA (OR: 1.24, 95% CI: 0.81–1.91, *I*^2^ = 0%) and resected OMA (OR: 1.40, 95% CI: 0.93–2.12, *I*^2^ = 0%) carried similar risks [93]. Additionally, compared to those without endometriosis, women who conceived spontaneously with endometriosis stage I/II (OR: 1.68, 95% CI: 1.20–2.35) and stage III/IV (OR: 1.72, 95% CI: 1.26–2.34) had higher risks for miscarriage [93]. Finally, early abortion (<12 weeks) risk was higher than late abortion risk in women with endometriosis (OR: 15.87, 95% CI: 8.12–31.03, *I*^2^ = 0%) [93]. In a similar manner, Zullo and investigators showed an increased risk for miscarriage in women with endometriosis (OR 1.75; 95% CI, 1.29–2.37) [94]. Horton et al. noticed an increased risk for miscarriage in both endometriosis and adenomyosis (OR 3.40, CI 1.41–8.65 and OR 1.30, CI 1.25–1.35, respectively) [92]. A cohort study with 24,667 Danish women with endometriosis, realized by Hjordt Hansen et al., discovered that there were 4.3 more ART-miscarriages (95% CI, 3.4–5.5) and 1.24 (95% CI, 1.2–1.29) more miscarriages in all endometriosis-patients [95]. Lalani et al. found maternal endometriosis was associated with higher risks for stillbirth (seven studies; OR = 1.29 [1.10, 1.52]) and neonatal death (three studies; OR = 1.78 [1.46–2.16]) [80]. Aris A. performed a study over a 12-year cohort in Eastern townships of Canada regarding adverse pregnancy outcomes and added to the general knowledge that endometriosis has a significant impact with a risk of fetal loss of 2.03 (95% CI, 1.42–2.90, *p* = 0.0001) including miscarriage (OR = 1.89; 95% CI, 1.23–2.93, *p* = 0.0052) and stillbirth (OR = 2.29; 95% CI, 1.24– 5.22, *p* = 0.012) [96]. Breintoft et al. reported a risk for the stillbirth of 1.27 95% (CI: 1.07–1.51) [37].

## 9. Gestational Diabetes Mellitus, Gestational Cholestasis

Pregnancy expresses association with insulin resistance (IR) and hyperinsulinemia that may predispose to the development of diabetes. Any degree of glucose intolerance with first recognition or onset during pregnancy defines gestational diabetes (GD) [97]. Intrahepatic cholestasis in pregnancy or gestational cholestasis (GC) is one of the most common hepatic conditions related to pregnancy. It usually appears during the third trimester and presents elevated alanine aminotransferase and/or bile acid, accompanied by pruritus. Clinical signs rapidly rectify after delivery [98]. Conti et al. conducted a cohort study and showed that 13.3% of women with endometriosis at first singleton pregnancy had a risk for GD (OR:2.13, 95% CI, 1.32–3.44) [84]. Lalani et al. found the risk for GD of 1.26 (95% CI, 1.03–1.55) and GC of 4.87 (95% CI, 1.85–12.83), respectively, but in the latter, only one study was reported [80]. Insufficient data are available so far to link these two conditions. Some researchers have tried to establish a link between endometriosis and GD [96,99], but newer information is needed.

## 10. Small for Gestational Age

Small for gestational age (SGA) is defined as the weight of the baby at birth that is less than 10th percentile for GA. Very often, SGA is used interchangeably with fetal growth restriction (FGR), but they are not synonymous [100]. Berlac et al. found an association between endometriosis mothers with SGA babies, with a high risk of 1.5 (1.4–1.6) [85]. Conversely, Horton found a very high risk for mothers with adenomyosis (OR: 3.90, CI 2.10–7.25, *p* < 0.001), but not for endometriosis [92]. In accordance with Berlac, Vigano and collaborators stated that not only primiparas but also multiparas with endometriosis presented high risk for SGA fetuses (OR: 2.72, 95% CI, 1.46–5.06) and (OR: 2.93, 95% CI, 1.28–6.67), respectively [101]. Vercellini et al. did not find any correlation between birth weight strata and SGA with endometriosis [75]. Conti and researchers found a very strong link between SGA babies and women with endometriosis at first pregnancy and discovered an incidence of 10.6%, OR: 2.72 (*p* = 0.002) [84]. More accurately, Breintoft et al. analyzed the risk separately for low birth weight, with pooled OR of 1.22 (95% CI: 0.99–1.49) and SGA with pooled OR of 1.18 (1.02–1.36), showing that the risk is high in women with endometriosis [37]. Conversely, Pérez-López also separated the risks, but he obtained different results: an increased risk for SGA babies (OR: 1.16; 95% CI: 1.05–1.28) and no risk for those with low birth weight and FGR [99]. Zullo et al. also stated that women with endometriosis had a higher risk for SGA fetuses (OR: 1.27; 95% CI: 1.03–1.57) [94].

## 11. Limitations and Further Perspectives

Endometriosis is an underdiagnosed disease worldwide. As is generally known, the delay in diagnosis is about seven years. Moreover, it is difficult to quantify and track adverse effects in patients with endometriosis since many give birth without confirmation of the diagnosis. The peritoneal cavity cannot be visualized in patients who deliver spontaneously. Moreover, patients with undetermined infertility or even with confirmed and possibly surgically treated endometriosis resort to IVF procedures before trying to conceive spontaneously for a year. Several variables cannot be tracked. One of them is the association of adenomyosis, which cannot be surgically treated in patients with the desire to procreate. Another variable is the symptomatic presence of pain, which is very subjective from patient to patient and does not correlate with the stage of the disease. Most studies on obstetric complications in patients with endometriosis come from assisted human reproduction clinics. Therefore, details about treatment and diagnostic manners are not always provided. Furthermore, microscopic lesions may remain even after thoroughly surgically treated endometriosis. The limitations from the current knowledge and literature are reflected in our review. More research is needed to shed light on this matter.

## 12. Conclusions

Endometriosis may negatively impact pregnancy outcomes, sharing some pathophysiologic mechanisms that lead to inflammation. Epigenetic alterations of a few genes (DLX5, CDKN1C, PTGER2, and GATA3) are common for endometriosis and obstetric complications.

This review supports the idea that women with endometriosis are at elevated risk for important adverse fetal, neonatal, and maternal outcomes. Maternal risks are higher for complications such as preeclampsia and placental issues. The newborns are at increased risk for being delivered preterm or having a higher neonatal death rate. Although ART is known to determine adverse outcomes of pregnancies, most studies have shown that endometriosis increases the risks of negative impact irrespective of the type of reproduction (spontaneous or using ART).

From a clinical view, women with endometriosis should benefit from increased surveillance during pregnancy to prevent neonatal and maternal complications.

Further investigations are mandatory to provide new insights into the common epigenetic pathways that underly interactions between obstetric complications and endometriosis.
